# Simultaneous resection of bilateral anomalous systemic supply to the basal segments of the lungs: a case report

**DOI:** 10.1186/s13019-015-0366-y

**Published:** 2015-10-31

**Authors:** Takashi Makino, Yoshinobu Hata, Hajime Otsuka, Satoshi Koezuka, Yoichiro Okubo, Kazutoshi Isobe, Naobumi Tochigi, Kazutoshi Shibuya, Sakae Homma, Akira Iyoda

**Affiliations:** 1 Division of Chest Surgery, Toho University School of Medicine, Tokyo, Japan; 2 Division of Respiratory Medicine, Toho University School of Medicine, Tokyo, Japan; 3 Department of Surgical Pathology, Toho University School of Medicine, Tokyo, Japan

**Keywords:** Surgery, Bilateral, Anomalous systemic supply to the basal segments of the lungs, Pulmonary sequestration

## Abstract

**Background:**

Anomalous systemic arterial supply to the normal basal lung segments is a sequestration spectrum variant (Pryce type 1) that is distinguished from pulmonary sequestration by normal bronchopulmonary and parenchymal tissues.

**Case presentation:**

A 33-year-old Japanese man was referred to our hospital because of an abnormal pulmonary shadow. Computed tomography showed two aberrant arteries arising from the descending aorta and running into the lower lung lobes on each side, without any bronchial anomaly. He was diagnosed with bilateral anomalous systemic supply to the basal segments. A left thoracotomy was performed and the aberrant arteries were ligated and dissected at their origin followed by left basal segmentectomy. Simultaneous right S10 segmentectomy was performed under video-assisted thoracic surgery.

**Conclusion:**

Although bilateral anomalous systemic arterial supply to the basal segments is extremely rare, knowledge of this anomaly should allow for a definitive diagnosis and appropriate therapy.

## Background

Anomalous systemic arterial supply to the normal basal lung segments [1] is a sequestration spectrum variant (Pryce type 1) that is distinguished from pulmonary sequestration by normal bronchopulmonary and parenchymal tissues [2, 3]. Surgery is indicated for this disease because of the potential risks including hemoptysis due to pulmonary hypertension and heart failure due to left-to-left shunt [4]. Although bilateral anomalous systemic arterial supply to the basal segments is extremely rare [5], knowledge of this anomaly allows for a precise diagnosis and ligation of the aberrant arteries followed by simultaneous dissection of the lesions on both sides.

## Case presentation

**Fig. 1:**
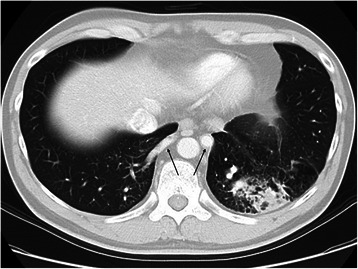
Computed tomography (CT) of the chest showed consolidation of the left lower lobe with no confined sequestrated area and two aberrant arteries (arrows)

**Fig. 2:**
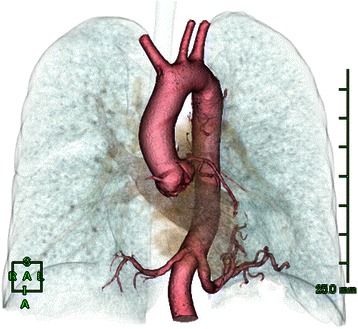
Three-dimensional CT revealed two aberrant arteries arising from the descending aorta and running into the lower lobes on each side, without a normal supply from the pulmonary artery

A 33-year-old Japanese man was referred to our hospital because of an abnormal pulmonary shadow found on a routine chest X-ray. He had suffered occasional hemoptysis for the past two years. A physical examination and laboratory investigation showed no remarkable findings. Computed tomography (CT) of the chest showed consolidation of the left lower lobe with normal bronchial tree and no confined sequestrated area (Fig. 1). Three-dimensional CT revealed two aberrant arteries arising from the descending aorta and running into the lower lobes on each side, without a normal supply from the pulmonary artery (Fig. 2). The right aberrant artery measured 7 mm in diameter, and the left aberrant artery was 13 mm in diameter. The bronchus and pulmonary vein appeared anatomically normal on the CT scan. These findings led us to the diagnosis of bilateral anomalous systemic supply to the basal segments of the lungs. Because simple occlusion or ligation of the anomalous artery had a risk of pulmonary infarction, we decided to remove the bilateral anomalous artery, and perform left basal segmentectomy and right S10 segmentectomy simultaneously.

**Fig. 3:**
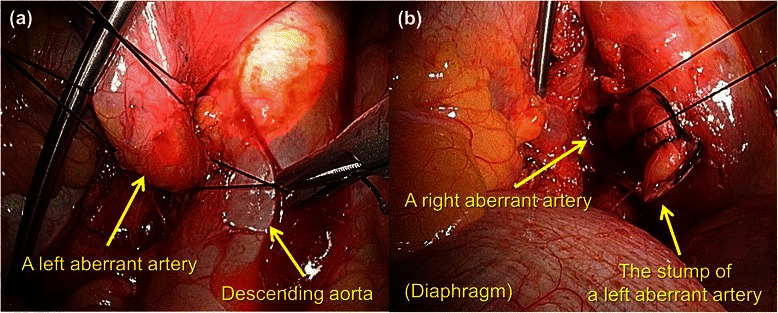
**a** A left aberrant artery arising from the descending aorta and running into the left lower lobe. **b** A right aberrant artery running into the right thoracic cavity and the stump of a resected left aberrant artery

He was initially placed in the right lateral decubitus position. A left thoracotomy was first performed to approach the site of origin of the two aberrant arteries. The aberrant artery to the left lower lobe was ligated and divided with an ATW 3.5-mm linear stapler (Ethicon Japan, Tokyo), and the aberrant artery to the right side was ligated at the descending aorta (Fig. 3). Then, a left basal segmentectomy was performed. A 24-French chest tube was placed, the lung was inflated under direct vision and the incisions were closed in layers. Subsequently, he was turned to the left lateral decubitus position. A right S10 segmentectomy was performed under video-assisted thoracoscopic surgery (VATS). The distal right aberrant artery was divided with an ATS 3.5-mm linear stapler. A 24-French chest tube was placed, and the incisions were closed in layers. His postoperative course was uneventful, and he was discharged from the hospital on the 9^th^ postoperative day. Microscopic examination of the resected specimen revealed atherosclerosis, intimal thickening and fibrosis in the both aberrant arteries, suggesting the local pulmonary hypertension. In the left lung parenchyma, macrophages containing hemosiderin were recognized, probably resulting from the pulmonary hemorrhage and consequent hemoptysis. There were findings of obstructive pneumonia in the left lung. The normal pulmonary artery which should exist, intra- or extralobar sequestrated lung parenchyma, or any other dysplastic changes could not be identified in any of the resected specimens. On the basis of these findings, pathologic evaluation of the resected tissue from both lungs confirmed the diagnosis of bilateral anomalous systemic supply to the basal segments of the lungs with left obstructive pneumonia. At the time of this report, 12 months after the resection, he was doing well.

## Discussion

Anomalous systemic arterial supply to the normal basal lung segments is a rare congenital anomaly typically involving the left lower lobe, where pulmonary arteries of the affected segments are also absent. This anomaly also is known as Pryce type 1 sequestration.

Bilateral pulmonary sequestration is extremely rare. Moreover, we were able to perform bilateral segmentectomy to preserve pulmonary function. Savic et al. [5] reported that there were only two bilateral intralobar sequestration cases in their evaluation of 540 pulmonary sequestration cases. To date, seven cases of a synchronous bilateral resection have been reported, and two of them were treated with synchronous video-assisted resection of bilateral pulmonary sequestration [6, 7]. However, those reports did not describe the operative procedures in detail. In the present case, we decided to first approach the site of origin of the aberrant arteries via a left thoracotomy; then, left basal segmentectomy and simultaneous right S10 segmentectomy were performed under VATS. We thought the ligation of the aberrant arteries at their origin was important because of the potential risk of aneurismal formation later.

Previous treatment for this disease was lobectomy or basal segmentectomy with anastomosis of the divided anomalous systemic artery to the pulmonary artery [2–4], or simple ligation of the anomalous artery [8]. Recently, coil embolization was reported in a few cases [9]. Although ligation and coil embolization could cause pulmonary infarction when the pulmonary arterial supply is absent, in most previous cases it has not led to severe complication because of the abundant collateral circulation from the bronchial, intercostal, inferior phrenic and the other nearby arteries [9]. In contrast, if collateral flow is not present, there is the potential risk of hemorrhage after simple ligation or embolization. In the present case, we decided to perform simultaneous resection of the bilateral anomalous systemic supply to the basal segments of the lungs because simple occlusion or ligation of the anomalous artery had a risk of pulmonary infarction and a risk of recurrent hemoptysis, although the long term data of the available therapeutic options have not been available until recently.

Evaluation of anomalous variations before selecting a therapeutic strategy and surgical procedure is essential. In the present case, we performed a left thoracotomy first according to the preoperative identification of two aberrant arteries on three-dimensional CT, which indicated that ligation of the two aberrant arteries could be performed from the left side.

## Conclusion

Although bilateral anomalous systemic arterial supply to the basal segments is extremely rare, knowledge of this anomaly should result in the prompt, careful tracing of the course of these vessels on CT, which should allow for a definitive diagnosis and selection of appropriate therapy.

## Informed consent

Written informed consent was obtained from the patient for publication of this Case report and any accompanying images. A copy of the written consent is available for review by the Editor-in-Chief of this journal.

## References

[1] Painter RL, Billig DM, Epstein I (1968). Anomalous systemic arterialization of the lung without sequestration. N Engl J Med..

[2] Hessel EA, Boyden EA, Stamm SJ, Sauvage LR (1970). High systemic origin of the sole artery to the basal segments of the left lung: findings, surgical treatment, and embryologic interpretation. Surgery..

[3] Iizasa T, Haga Y, Hiroshima K, Fujisawa T (2003). Systemic arterial supply to the left basal segment without the pulmonary artery: four consecutive cases. Eur J Cardiothorac Surg..

[4] Yamanaka A, Hirai T, Fujimoto T, Hase M, Noguchi M, Konishi F (1999). Anomalous systemic arterial supply to normal basal segments of the left lower lobe. Ann Thorac Surg..

[5] Stern R, Berger S, Casaulta C, Raio L, Abderhalden S, Zachariou Z (2007). Bilateral intralobar pulmonary sequestration in a newborn, case report and review of the literature on bilateral pulmonary sequestrations. J Pediatr Surg..

[6] Yamamura Y, Hida Y, Kaga K, Kawada M, Niizeki H, Ichinokawa M (2009). Simultaneous resection of bilateral intralobar and extralobar pulmonary sequestrations with video-assisted thoracoscopic surgery. Ann Thorac Surg..

[7] Morse CR, Ishitani MB, Cassivi SD (2006). Video-assisted resection of bilateral intralobar pulmonary sequestrations. J Thorac Cardiovasc Surg..

[8] Baek WK, Cho J, Kim JT, Yoon YH, Kim KH, Lim HK (2006). Systemic arterial supply to normal basal segments of the left lower lobe along with the pulmonary artery: is lung resection warranted?. J Thorac Cardiovasc Surg..

[9] Jiang S, Shi JY, Zhu XH, Chen C, Sun XW, Yu D (2011). Endovascular embolization of the complete type of anomalous systemic arterial supply to normal basal lung segments: a report of four cases and literature review. Chest..

